# Effectiveness of a perforated spoon for reducing salt intake in a university cafeteria

**DOI:** 10.3389/fpubh.2026.1845807

**Published:** 2026-06-18

**Authors:** Aya Murayama, Aimi Hamasu, Ikumi Tabata, Manami Sato, Eri Tajiri, Nana Nakashima, Tatsuaki Sakamoto

**Affiliations:** Faculty of Environmental & Symbiotic Sciences, Prefectural University of Kumamoto, Kumamoto, Japan

**Keywords:** cafeteria setting, nudge, perforated spoon, randomized crossover trial, salt reduction, ramen

## Abstract

**Objective:**

This study investigated the salt reduction effect of using a perforated spoon to eat ramen noodles.

**Design:**

Randomized crossover.

**Setting:**

University cafeteria.

**Participants and procedures:**

A total of 39 male and 38 female university students participated in the study. Participants consumed ramen under two conditions: (1) using chopsticks and a regular spoon (‘regular spoon condition') and (2) using chopsticks and a perforated spoon (‘perforated spoon condition'). Soup intake, salt intake, and subjective ratings of hunger, fullness, and palatability were compared between conditions. After excluding those who withdrew, 35 male and 34 female students were included in the final analysis. Sex-specific comparisons were conducted.

**Results:**

Among male participants, the median (25th, 75th percentiles) soup intake was 95.0 (77.0, 236.0) g with the perforated spoon and 141.0 (93.0, 347.0) g with the regular spoon (*P* = 0.004); salt intake was 2.4 (2.1, 4.9) g and 3.1 (2.4, 6.9) g (*P* = 0.022), respectively. Among female participants, soup intake was 84.5 (71.0, 122.0) g and 129.5 (95.5, 165.3) g (*P* < 0.001), and salt intake was 2.2 (1.9, 2.8) g and 3.0 (2.4, 3.6) g (*P* < 0.001), respectively. Post-meal satiety and palatability did not differ significantly between the two conditions (*P* > 0.05).

**Conclusions:**

Using a perforated spoon during ramen consumption significantly reduced soup and salt intake in both sexes without compromising satiety or palatability. These findings suggest that providing perforated spoons as standard utensils in institutional cafeterias may support salt-reduction efforts, although further validation in other populations and settings is needed.

## 1 Introduction

Excessive dietary sodium intake is a known risk factor for hypertension, cardiovascular disease (CVD), stroke, kidney disease, and cardiovascular mortality worldwide (1) In response, the World Health Organization (WHO) recommends limiting daily salt intake to less than 5 g (approximately 2 g sodium) to reduce blood pressure and prevent CVD (2). Despite these guidelines, global salt intake often exceeds 7–10 g per day, particularly in East Asia, where traditional diets and processed or restaurant foods contribute significantly to sodium consumption (3).

To translate the global challenge of excessive sodium intake into actionable targets, it is important to identify commonly consumed foods that deliver a high and concentrated sodium load. In East Asia, ramen represents such a target. It is relatively frequently consumed in Japan, particularly at lunch (4), and typically contains a large amount of sodium per serving; a single restaurant-style bowl in Japan has been reported to contain approximately 6.5 g of sodium chloride (5), which can exceed the WHO's recommended daily limit in one meal. Importantly, most of this sodium is concentrated in the soup, making soup consumption a key driver of total intake (6). This characteristic also makes ramen especially amenable to behavioral modification through utensil-based interventions that can reduce sodium intake without altering the food itself. Similar concerns have been raised in other Asian countries, such as South Korea and China, where instant noodles are widely consumed (7, 8). Given that consuming all the soup substantially increases sodium intake, leaving the soup behind has been recommended as a practical strategy for reducing salt intake (9).

However, efforts to reduce salt intake are often limited due to low levels of dietary awareness and motivation. The 2019 National Health and Nutrition Survey in Japan found that a substantial segment of the population showed little interest in improving their eating habits (10). To address this challenge, behavioral “nudge” interventions have gained attention. These strategies aim to influence eating behavior subtly through environmental modifications, such as changes to tableware, without relying on active decision making (11–14).

A recent laboratory study in Japan demonstrated that the use of a perforated spoon when consuming ramen reduced soup and salt intake in male university students (15). However, findings from controlled settings may not fully translate to everyday eating contexts as participants may adjust their behavior when they are aware that they are being observed or measured (16, 17). Moreover, sex-based differences in eating behaviors, including eating speed, salt taste sensitivity, and attitudes toward salt, have been reported (18, 19), underscoring the need for more inclusive evaluations in settings that better reflect routine dining conditions.

Importantly, the present study focuses on modifying how foods are consumed rather than what is being served. Perforated spoons represent a low-cost, scalable, and operationally simple intervention that institutional food service providers can implement with minimal burden. While upstream strategies, such as food reformulation, reducing sodium content at the source, or portion size modification, are generally considered more effective and equitable for achieving population-level salt reduction, as suggested by systematic reviews demonstrating an effectiveness hierarchy of salt reduction interventions (20), utensil-based approaches may serve as complementary, downstream behavioral nudges. Such strategies can support salt reduction efforts by influencing eating behavior without requiring sustained individual behavioral change or structural modifications to the food supply.

Therefore, in this study, we aimed to evaluate the effectiveness of perforated spoons in reducing salt intake during ramen consumption among male and female university students in a semi-naturalistic cafeteria setting. We also examined subjective outcomes such as satiety and palatability to assess the practical applicability of this approach as a feasible strategy to support salt reduction in institutional dining environments.

## 2 Methods

### 2.1 . Experimental method

#### 2.1.1 . Participants

The sample size (*n* = 34) was calculated using G^*^Power 3.1.9.2 software (Heinrich-Heine-Universität Düsseldorf, Düsseldorf, Germany) based on previous studies (15) indicating an expected salt reduction of 1.0 ± 2.0 g with the use of a perforated spoon (α = 0.05, power = 0.8). The calculated effect size (Cohen's d) was 0.5. To enable sex-specific analyses, recruitment was conducted with the aim of obtaining 34 participants of each sex, and additional participants were recruited to account for potential dropouts. Given the nature of the study, the true objective was withheld from the participants to minimize demand characteristics; they were informed that the study was concerned with the palatability of ramen. During recruitment, the participants were informed that the study involved three experimental ramen meals and that questionnaires would be administered before and after each meal to assess palatability.

Students who met the following inclusion criteria were recruited: (1) those not currently following any dietary restrictions for weight loss or salt reduction, and (2) non-smokers. The authors provided individual explanations of the study on campus to 39 of 44 male university students and 38 of 45 female university students who consented to participate. As part of the ethical considerations, all participants were informed that their cooperation was voluntary and that they could withdraw their consent at any time, even after agreeing to participate. Written informed consent was obtained from all participants in advance. The study protocol was approved by the Ethics Committee of the Prefectural University of Kumamoto.

#### 2.1.2 . Study design

This study used a randomized crossover design.

#### 2.1.3 . Study procedures

**Figure 1:**
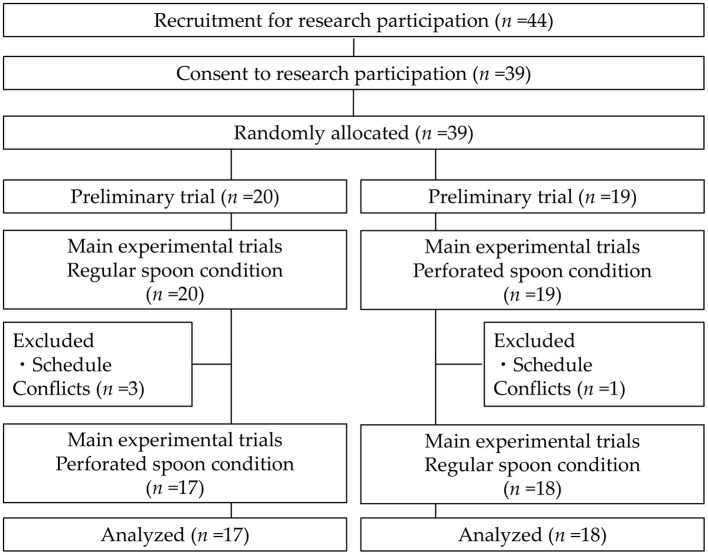
Flow diagram illustrating the process of male student participant selection in each randomized crossover trial.

**Figure 2:**
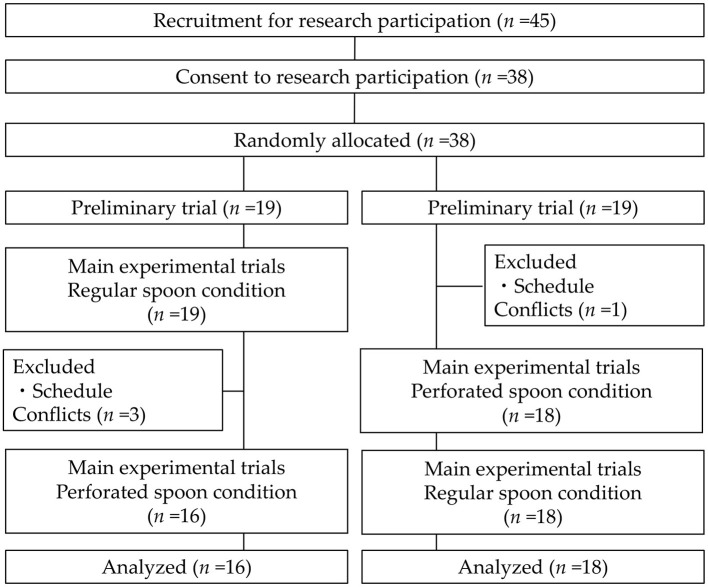
Flow diagram illustrating the process of female student participant selection in each randomized crossover trial.

The study procedure followed that of previous studies (15) and is shown in Figures 1, 2. All trials, including the preliminary trial, were conducted from April to November 2024, excluding August and September, during the university's long vacation period. Each participant participated in three trials: one preliminary and two main trials. All trials were conducted during lunchtime (11:50–12:50), with an interval of at least 7 days between each trial. In the preliminary trial, participants were instructed to consume a test meal using chopsticks and a regular spoon. This preliminary trial was conducted to familiarize participants with the study procedures and environment, thereby minimizing variability due to unfamiliarity. It was not intended to standardize dietary intake. The two main experimental trials consisted of: (1) consuming the test meal with chopsticks and a regular spoon (hereafter referred to as the “regular spoon condition”), and (2) consuming the test meal with chopsticks and a perforated spoon (hereafter referred to as the “perforated spoon condition”).

Participants completed two main trials (regular spoon condition and perforated spoon condition) in a randomized crossover design. The preliminary trial and two main trials were separated by at least 7 days to minimize potential carryover effects. For the two main trials, participants were assigned to one of two sequence groups: regular spoon followed by perforated spoon, or perforated spoon followed by regular spoon. The allocation sequence was generated by the principal investigator (TS) using a random number generator. Participants were assigned to one of the two sequence groups (regular spoon followed by perforated spoon, or the reverse), with allocation implemented to ensure balanced group sizes. We also examined potential sequence effects analytically and found no significant influence of condition order on the outcomes.

To minimize potential behavioral changes arising from awareness of the study purpose, participants were not informed of the specific aim of the study prior to participation. This approach was intended to reduce reactivity during the meal. However, the investigators who conducted the experiments and collected the data were not blinded to the study conditions.

Participants, including those in the preliminary trial, were instructed to refrain from moderate or strenuous exercise from the day before the study until the start of the study, and to maintain their usual routines. On the days of the main experimental trials, participants were instructed to replicate their habitual breakfast and morning activities as observed on the day of the preliminary trial. Some participants typically ate breakfast, while others did not. Those who ate breakfast on the day of the preliminary trial were instructed to do so on the days of the two main trials, with no specific instructions regarding breakfast type. Participants who did not habitually consume breakfast were asked to attend the study in a fasted state. Thus, the morning routine was not standardized across participants but was replicated within each participant.

All trials were conducted in the cafeteria of the Prefectural University of Kumamoto (325 seats, total floor area of 1,190 m^2^), where participants were assigned designated seats for their meals. At lunchtime, approximately 80% of the seats in the student cafeteria were occupied. Seating arrangements ensured that participants were not seated next to each other. The cafeteria served as a regular dining environment used by other students for lunch. Before eating the test meal, participants were asked to report their subjective evaluations, such as hunger, as well as details of their morning activities, including breakfast consumption, snacking, and physical activity. Participants were given standardized instructions to “eat ramen as usual.” No instructions were provided on the use of the spoon. After meals, participants were asked about their subjective appetite.

As observation may affect food consumption and eating speed (16), participants were not monitored or recorded during their meals, and the researchers waited in a location hidden from view. After participants finished eating, they were asked to clear their trays, and the researchers collected the dishes and utensils. Following the meal, the participants were asked about their subjective sensations, including fullness.

#### 2.1.4 . Test meal

The test meal consisted of a ramen noodle dish comprising wheat noodles, sliced pork, seaweed, bean sprouts, and other soup ingredients. According to a study examining the food-based dietary patterns of Japanese people during breakfast, lunch, and dinner, ramen is frequently consumed during lunch (4).

The nutritional details of the test meals are presented in Table 1. The energy equivalent of the test meal ramen was 451 kcal, and the salt equivalent was 7.1 g (0.8 g for the noodles and ingredients, and 6.3 g for the soup). The ramen soup was flavored using a commercial product [Ramen Soup (Chicken Stock & Soy Sauce), EON CO, Chiba, Japan]. The salt equivalent of the noodles was calculated by multiplying the weight of the noodles by the salt content ratio (0.2%) when the noodles were boiled. Drinking water was served in 300-mL portions at the same time as the test meal.

**Table 1:** Test meal: Ramen ingredients and nutritional value.

Food items	Weight (g)	Energy (kcal)	Protein (g)	Fat (g)	Carbohydrate (g)	Salt equivalent^*^(g)
Wheat noodles (boiled)	209.0	311.0	10.2	2.5	61.0	0.4
Ramen soup	348.0	80.0	4.7	5.4	3.3	6.3
Sliced pork	14.0	50.0	1.6	4.6	0.6	0.4
Bean sprouts	50.0	6.0	0.9	0.1	1.3	0.0
Green onion	5.0	3.0	0.1	0.0	0.3	0.0
Seaweed	0.5	1.0	0.2	0.0	0.2	0.0
Total	626.5	451	17.7	12.6	66.7	7.1

^*^The salt equivalent of noodles was the amount of salt after boiling the noodles.

#### 2.1.5 . Spoons

**Figure 3:**
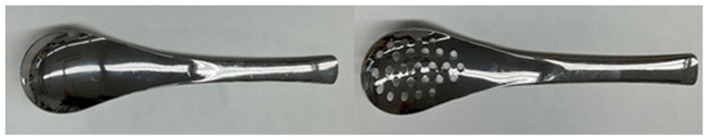
Regular spoon and perforated spoon.

The regular and perforated spoons used in this study were identical to those used in a previous study (15) (Figure 3). The lengths of the tips of the regular spoon (Shimomura Kihan Co., Niigata, Japan) and perforated spoon (Shimomura Kihan Co., Niigata, Japan) were 180 mm and 177 mm, respectively, with both spoons having a width of 37 mm. The perforated spoon had 27 holes. The regular spoon could hold approximately 9.0 g of soup per scoop, whereas the perforated spoon allowed the soup to drain through the holes, enabling only solid ingredients to be scooped.

### 2.2 . Survey items

#### 2.2.1 . Physical measurements

During the preliminary test, participant height was measured to the nearest 0.1 cm, and weight was measured to the nearest 0.1 kg using a digital scale (TBF-102; Tanita Corporation, Tokyo, Japan). Body mass index was calculated as weight (kg) divided by height squared (m^2^).

#### 2.2.2 . Subjective evaluation

Visual analog scales ranging from 0 to 10.0 cm were used to assess participants' appetites (hunger and fullness) before and after meals. For hunger, the right end of the scale was labeled ‘very hungry' and the left end ‘not hungry at all,' with participants marking their response on a 10.0 cm line. The visual analog scale scores were measured to the nearest 0.1 cm. The same method was used to evaluate the taste of the ramen, with the right end labeled ‘very delicious' and the left end ‘not delicious at all.' Higher scores (closer to 10.0) indicated greater hunger, fullness, and perceived palatability (better taste).

#### 2.2.3 . Salt intake, quantity of the meal consumed, and water consumption

The amount of salt intake and quantity of the meals consumed were calculated by subtracting the weight of the remaining noodles, ingredients, and soup (g) from the initial weight provided (g). Water intake was calculated by subtracting the remaining water weight from the initial amount provided.

### 2.3 . Statistical analyses

All analyses were conducted separately for male and female participants. To compare paired individual results between the two spoon conditions, the Wilcoxon signed-rank test was used for subjective evaluations, salt intake, and quantity of the meals consumed. Nonparametric tests were applied for salt and soup intake because normality could not be confirmed using histograms and the Shapiro–Wilk test (*P* < 0.05). The Wilcoxon effect size r (r = Z / √N) was calculated to compare the effect size of each outcome (21). To assess potential sequence effects, intra-participant differences between conditions (regular spoon condition – perforated spoon condition) were calculated for each participant. These differences were then compared between the two sequence groups (regular → perforated vs. perforated → regular) using the Mann–Whitney U test. All statistical analyses were performed using SPSS Statistics (version 27.0; IBM Corp., Armonk, NY, USA), with the significance level set at 5% (two-tailed).

## 3 Results

### 3.1 Participant characteristics

Of the 39 male and 38 female participants who consented to participate in the study, four men and four women withdrew owing to scheduling conflicts. Hence, 35 men and 34 women were included in the final analysis. Table 2 presents the physical characteristics of the participants.

**Table 2:** Participants' physical characteristics.

Characteristics	Male (*n* = 35)		Female (*n* = 34)	
	mean	SD	mean	SD
Age (years)	19.7	1.1	20.4	1.2
Height (cm)	172.2	4.9	159.4	5.4
Weight (kg)	64.0	10.0	50.1	5.5
Body mass index	21.6	3.3	19.7	1.7

SD, standard deviation.

### 3.2 Comparison of subjective evaluations before and after eating under both spoon conditions

Table 3 presents the results of the comparison of subjective evaluations before and after meals. There were no significant differences in hunger or fullness before and after meals between the two-spoon conditions in either male or female participants (*P* > 0.05). Additionally, there was no significant difference in the palatability of the test meals between male and female participants (*P* > 0.05).

**Table 3:** Comparison of subjective evaluations before and after eating under both spoon conditions.

Variable	Before meals		*P* ^*^	After meals		*P* ^*^
Male (*n* = 35)						
Hunger (cm)						
Regular spoon	8.0	(5.0, 8.5)	0.982	1.3	(0.5, 2.0)	0.088
Perforated spoon	7.5	(5.0, 8.5)		1.7	(1.0, 2.0)	
Fullness (cm)						
Regular spoon	2.0	(1.0, 5.0)	0.712	8.5	(8.0, 9.5)	0.801
Perforated spoon	2.0	(1.0, 3.5)		8.8	(8.0, 9.3)	
Palatability (cm)						
Regular spoon				9.0	(8.0, 9.8)	0.308
Perforated spoon				9.0	(7.5, 9.6)	
Female (*n* = 34)						
Hunger (cm)						
Regular spoon	7.6	(6.4, 9.0)	0.868	1.0	(0.0, 2.1)	0.864
Perforated spoon	8.0	(6.0, 8.6)		1.0	(0.0, 2.0)	
Fullness (cm)						
Regular spoon	2.0	(1.0, 3.4)	0.873	9.0	(8.0, 9.7)	0.483
Perforated spoon	2.0	(0.9, 3.0)		9.0	(8.0, 10.0)	
Palatability (cm)						
Regular spoon				9.4	(8.6, 10.0)	0.719
Perforated spoon				9.1	(8.5, 10.0)	

Values: median (25th and 75th percentile values). Satiety (hunger and fullness) and palatability were assessed by subjective psychological ratings using a 0–10-cm visual analog scale. The visual analog scale was evaluated in increments of 0.1 cm. Ratings closer to 10 indicated more hunger, fullness, and palatability (better taste). ^*^Wilcoxon signed-rank test.

### 3.3 . Comparison of salt intake, quantity of the meal consumed, meal time, and related variables under both spoon conditions

The results of the comparisons of salt intake, meal time, and related variables under both spoon conditions are presented in Table 4. Among male participants, the median soup intake (25th, 75th percentiles) g was 95.0 (77.0, 236.0) g under the perforated spoon condition, compared with 141.0 (93.0, 347.0) g under the regular spoon condition (*P* = 0.004, *r* = 0.490). Correspondingly, the salt intake from the whole meal was 2.4 (2.1, 4.9) g with the perforated spoon and 3.1 (2.4, 6.9) g with the regular spoon (*P* = 0.022, *r* = 0.386). These effect sizes indicate a moderate reduction in soup and salt intake.

**Table 4:** Salt intake, quantity of the meal consumed, and quantity of water consumed under each spoon condition in male and female participants.

Variable	Regular spoon condition		Perforated spoon condition		*P* ^*^	*r^†^*
Males (*n* = 35)						
Salt intake						
Whole meal (g)	3.1	(2.4, 6.9)	2.4	(2.1, 4.9)	0.022	0.386
Ramen soup (g)	2.5	(1.7, 6.3)	1.7	(1.4, 4.2)	0.006	0.467
Noodles and ingredients (g)	0.7	(0.6, 0.7)	0.7	(0.6, 0.7)	0.851	0.032
Quantity of the meal consumed						
Whole meal (g)	388.0	(350.0, 592.0)	347.0	(326.0, 499.0)	0.005	0.479
Ramen soup (g)	141.0	(93.0, 347.0)	95.0	(77.0, 236.0)	0.004	0.490
Noodles and ingredients (g)	248.0	(242.0, 258.0)	253.0	(237.0, 265.0)	0.743	0.055
Quantity of water consumed (g)	297.0	(186.0, 300.0)	295.0	(196.0, 299.0)	0.270	0.187
Females (*n* = 34)						
Salt intake						
Whole meal (g)	3.0	(2.4, 3.6)	2.2	(1.9, 2.8)	< 0.001	0.664
Ramen soup (g)	2.3	(1.7, 3.0)	1.5	(1.3, 2.2)	< 0.001	0.679
Noodles and ingredients (g)	0.6	(0.6,0.7)	0.6	(0.6, 0.7)	0.221	0.210
Quantity of the meal consumed						
Whole meal (g)	377.0	(342.5, 403.8)	329.0	(313.5, 364.8)	< 0.001	0.688
Ramen soup (g)	129.5	(95.5, 165.3)	84.5	(71.0, 122.0)	< 0.001	0.682
Noodles and ingredients (g)	246.5	(237.5, 256.0)	243.0	(235.0, 254.3)	0.209	0.216
Quantity of water consumed (g)	172.5	(108.8, 297.0)	151.0	(93.8, 252.8)	0.668	0.074

Values: median (25th and 75th percentile values). ^*^Wilcoxon signed-rank test. ^†^The Wilcoxon effect size (*r* = Z / √N).

Among female participants, the median soup intake was 84.5 (71.0, 122.0) g under the perforated spoon condition, which was significantly lower than 129.5 (95.5, 165.3) g under the regular spoon condition (*P* < 0.001, *r* = 0.682). Similarly, the salt intake from the whole meal was 2.2 (1.9, 2.8) g with the perforated spoon, compared with 3.0 (2.4, 3.6) g with the regular spoon (*P* < 0.001, *r* = 0.664). These effect sizes indicate a large reduction in soup and salt intake.

Overall, both male and female participants showed significantly lower soup and salt intake under the perforated spoon condition than under the regular spoon condition. No significant sequence effects were observed in either male or female participants.

Regarding complete soup consumption, 10 male participants consumed all the soup under the regular spoon condition, whereas seven did so under the perforated spoon condition. All seven male participants (20.0%) who completely consumed the soup under the perforated spoon condition also completely consumed the soup under the regular spoon condition. One male participant consumed soup only with the perforated spoon. Two female participants (5.9%) who completely consumed the soup under the perforated spoon condition also consumed the soup under the regular spoon condition. None of the female participants consumed all of the soup only under the perforated spoon condition. In both male and female participants, no significant differences in water intake were observed between the two spoon conditions (*P* > 0.05).

## 4 Discussion

In the current study the salt reduction effect of using a perforated spoon during ramen consumption was investigated in an everyday university cafeteria setting, using analyses stratified by sex. The results showed that both male and female participants exhibited significant reductions in soup and salt intake under the perforated spoon conditions, with moderate effect sizes in male participants and large effect sizes in female participants. Furthermore, there were no significant differences in the after-meal ratings of satiety or palatability between the two conditions. These findings extend previous laboratory-based evidence to semi-naturalistic cafeteria setting and underscore the practical potential of a simple, low-cost utensil modification as a public health intervention.

The observed reduction in salt intake can be attributed to the decreased amount of soup consumed when using the perforated spoon, which limited the ability to scoop up the soup. Specifically, the median soup intake under the perforated spoon condition was approximately 45 g less than that under the regular spoon condition in male and female participants, corresponding to approximately five spoonful's with the regular spoon. Consequently, salt intake decreased from 3.1 g to 2.4 g in male participants and from 3.0 g to 2.2 g in female participants, representing an approximate 20% reduction in both sexes. These reductions corresponded to moderate effect sizes in male participants and large effect sizes in female participants, supporting the practical relevance of the findings.

A previous study (15) conducted under laboratory conditions using a similar method in male participants reported a median soup intake of 97.5 g under the regular spoon condition and 65.5 g under the perforated spoon condition. In contrast, in the present study conducted in the university cafeteria, median soup intake among male participants was 141.0 g under the regular-spoon condition and 95.0 g under the perforated spoon condition. Laboratory settings may alter eating behavior compared with habitual situations (16). In the present study, the influence of observation was minimized by not monitoring participants during their meals and by replicating an environment similar to their usual dining context. Therefore, the observed results likely reflect participants' habitual eating behaviors more accurately.

Overall, a salt reduction effect associated with the use of a perforated spoon was observed among the participants. However, a subset of participants [seven males (20.0%) and two females (5.9%)] drank all the soup in the perforated spoon condition. As participants were not directly observed during meals, the underlying mechanism cannot be verified. While it is possible that some participants consumed the soup directly from the bowl, this behavior cannot be considered universal and is not necessarily common among the majority; hence, this interpretation should be regarded as speculative rather than empirically confirmed. Importantly, in this study, sodium intake was estimated based on the total amount of soup consumed, regardless of the method of consumption; therefore, differences in consumption method would not affect the measurement of soup intake or the estimated sodium intake. In Japan, drinking soup directly from the bowl is not generally considered poor table manners and may be observed in some contexts, which could partly explain the limited effectiveness of the utensil-based nudge for these individuals. These findings suggest that utensil-based nudges may be less effective for a subset of individuals with strong habitual consumption patterns, and that complementary strategies—such as improving salt-related nutrition literacy or raising awareness of sodium-related health risks—may be warranted (22, 23).

A key implication of this study is the feasibility of implementing perforated spoons as the default utensil in institutional dining settings. As a low-cost and operationally simple intervention, they require no additional decision-making or behavioral effort from consumers, aligning with public health strategies that reduce cognitive burden and promote healthier default choices. Furthermore, previous research has shown that subtle changes in serving utensils, such as replacing spoons with tongs, can reduce food intake by increasing the effort required to serve food, thereby promoting healthier food choices (13). The accumulation of such utensil-based and environmental modifications may contribute to the development of dining environments that promote healthier eating behaviors.

The current study had some limitations. First, the participants were limited to university students in a single semi-naturalistic cafeteria setting; therefore, the findings may not be generalizable to other populations or settings. In particular, the effectiveness and acceptability of the intervention may differ in older adults, non-student populations, and other food-service environments. Previous research has indicated that salt preferences and perceptions differ according to age (24, 25). Second, this study only evaluated the short-term effects of the intervention; hence, the long-term impact on salt reduction remains unknown. Third, although the study was conducted in a semi-naturalistic cafeteria setting, participants were aware of their involvement in the study, which may have influenced their eating behavior (i.e., reactivity). Fourth, all participants completed a preliminary trial prior to the main trials to familiarize themselves with the study procedures. Although this was intended to reduce variability due to unfamiliarity, this prior exposure may have influenced subsequent eating behavior through learning or expectation effects and cannot be entirely excluded as a source of bias. Fifth, variation in breakfast composition was not controlled, which may have influenced hunger levels or soup intake at lunch.

Despite these limitations, the present findings suggest that using a perforated spoon during ramen consumption can reduce soup intake and achieve a salt reduction effect even in a semi-naturalistic cafeteria setting. The salt reduction per meal (approximately 0.7–0.8 g) represents a proportion of the total salt content of a typical ramen meal and may contribute to achieving recommended daily salt intake targets if sustained.

While upstream strategies such as food reformulation and portion size reduction remain central to population-level salt reduction, this intervention may be viewed as a complementary, low-burden behavioral nudge. Given their low cost, scalability, and minimal operational burden, integrating perforated spoons into university or workplace cafeterias may represent a practical environmental strategy to support salt reduction. By embedding such unobtrusive nudges into everyday dining environments, institutional food service providers can promote healthier eating patterns without compromising the consumer experience, advancing public health goals related to salt reduction.

## 5 Conclusions

In this study, we examined the salt-reduction effect of using a perforated spoon during ramen consumption among university students in a semi-naturalistic cafeteria setting. The results demonstrated that using a perforated spoon reduced soup intake and achieved a salt reduction effect in both male and female participants. These findings suggest that a simple utensil-based modification may represent a practical approach to support salt reduction in institutional dining environments. However, further studies are needed to evaluate the effectiveness, acceptability, and sustainability of this intervention in other populations and settings.

## Data Availability

The raw data supporting the conclusions of this article will be made available by the authors, without undue reservation.
